# Epidemiological Profile of Retinopathy of Prematurity in Central India

**DOI:** 10.7759/cureus.87259

**Published:** 2025-07-03

**Authors:** Rajesh S Joshi, Harsha Singh, Riya Joshi, Pradeep Tekade, Abhijit S Patil

**Affiliations:** 1 Ophthalmology, Government Medical College, Akola, IND; 2 Ophthalmology, Government Medical College and Hospital, Nagpur, IND; 3 Ophthalmology, Datta Meghe Medical College, Nagpur, IND; 4 Ophthalmology, Malati Multispeciality Hospital and Medical College, Akola, IND

**Keywords:** blindness due to retinopathy of prematurity, central india, oxygen therapy in rop, premature deliveries, retinopathy of prematurity, vasoproliferative disorder

## Abstract

Background: Retinopathy of prematurity (ROP) is a vasoproliferative retinal disorder predominantly affecting premature infants with low birth weight. This study aims to investigate the incidence, risk factors, and clinical outcomes of ROP among preterm infants in central India, where the healthcare infrastructure and accessibility vary significantly.

Materials and methods: This prospective, observational, non-randomized study was conducted over three years in a tertiary teaching hospital in central India. The study included neonates with a gestational age of less than 34 weeks and/or birth weight of below 2000 grams, as well as neonates between 34 and 36 weeks of gestation who had specific risk factors (e.g., oxygen therapy, sepsis, multiple births, blood transfusion) and were admitted to the neonatal intensive care unit. Infants were screened for ROP, and outcomes were categorized based on the severity of the disease.

Results: Of the 300 preterm infants enrolled, 90 (30%) developed ROP, with 60 (20%) having mild ROP and 30 (10%) requiring treatment for severe ROP. Severe ROP was significantly associated with lower gestational age (mean: 29.8 weeks) and lower birth weight (mean: 1200 grams). Prolonged oxygen therapy, sepsis, multiple pregnancies, and blood transfusions were identified as significant risk factors. Following laser photocoagulation treatment, 85% (n=25) of infants with severe ROP showed regression, though 15% (n=13) experienced complications leading to significant visual impairment.

Conclusion: This study emphasizes the importance of comprehensive ROP screening, optimized oxygen therapy guidelines, and robust infection control to mitigate the risk of ROP in central India. Future research should focus on long-term outcomes and innovative prevention and treatment approaches tailored to this population's unique needs.

## Introduction

Retinopathy of prematurity (ROP) is a vasoproliferative disorder of the retina that predominantly affects premature infants with low birth weight. ROP remains a significant cause of childhood blindness globally, particularly in developing countries where neonatal care is improving, yet disparities in healthcare infrastructure persist. The pathogenesis of ROP is multifactorial, involving both genetic predisposition and environmental factors such as oxygen therapy and neonatal intensive care practices. ROP is a disease with a wide spectrum, ranging from mild, transient changes in the retina with regression to severe progressive vasoproliferation, fibrosis, and retinal detachment, leading to blindness. Asian countries account for 40% of the world's preterm births [1]. The rising trend has been observed in developing countries, including India [2]. Despite advancements in neonatal care, the incidence of ROP varies widely across different regions, influenced by differences in socioeconomic status, accessibility to healthcare services, and variations in clinical practices. Central India, characterized by a diverse population and varying levels of healthcare accessibility, presents a unique landscape for studying the epidemiology of ROP. Previous studies on ROP in India have primarily focused on metropolitan areas, leaving a gap in the understanding of ROP in central India's semi-urban and rural settings. The identification of region-specific risk factors and outcomes is crucial for developing targeted prevention and management strategies. Therefore, this study aims to investigate the incidence, risk factors, and clinical outcomes of ROP among preterm infants in central India. By comprehensively analyzing the demographic and clinical profiles of affected infants, this research seeks to contribute to the existing body of knowledge and inform healthcare policies to mitigate the burden of ROP in this region. Understanding the local epidemiology of ROP will facilitate early detection, timely intervention, and, ultimately, the reduction of visual impairment in this vulnerable population.

## Materials and methods

The study was conducted after obtaining approval from the Institutional Ethical Committee of Government Medical College, Nagpur, India (approval number: N/A), and it adhered to the Declaration of Helsinki. It was a prospective, observational, non-randomized, hospital-based study conducted at Government Medical College and Hospital, Nagpur, Maharashtra, India, over a period of three years (May 2021-May 2024). We adopted a consecutive sampling approach, including all eligible preterm infants admitted to the neonatal intensive care unit (NICU) between May 2021 and May 2024. This approach ensured maximal inclusion and reduced selection bias, providing a sufficiently large cohort to estimate ROP incidence and assess associated risk factors within this regional context. Written informed consent was taken from the parents/guardians to participate in the study. The study population included the following: neonates with a gestational age of less than 34 weeks, neonates with a birth weight of less than 2000 grams, and neonates between 34 and 36 weeks of gestation who had specific risk factors and were admitted to the NICU. These risk factors included hyperglycemia, hypoglycemia, oxygen therapy (via any route and for any duration), blood component transfusion, multiple gestation, maternal or neonatal anemia, sepsis, respiratory distress syndrome, and neonatal jaundice. These criteria were based on the National Neonatology Forum (NNF) India guidelines for ROP screening [3]. Infants who were lost to follow-up, babies with ocular and retinal disorders that hinder fundus evaluation, and infants with incomplete data on gestational age and birth weight were excluded from the study. All the newborns included in the study underwent thorough evaluation. The evaluation included gestational age at birth, sex, weight of the baby, and mode and duration of oxygen therapy given to the neonate (through either nasal prongs, nasal continuous positive airway pressure (CPAP), or mechanical ventilation). During the study period, oxygen blenders were not routinely available, and in most cases, oxygen was delivered via standard flow meters without precise FiO₂ titration. The concentration of oxygen administered was typically in the range of 40-60%, particularly in the early postnatal period. Although saturation targets were generally maintained between 90% and 95%, real-time blending and monitoring were not consistently feasible. A thorough clinical and neurological examination was performed. A general neurological examination was performed by the attending neonatologist as part of routine NICU assessment. This was not used to assess direct risk for ROP but was included to evaluate overall neonatal health and to identify any comorbidities that could impact follow-up or systemic prognosis. We do not recommend neurological examination as a standard component of ROP screening. Laboratory investigations included complete blood count, blood culture, C-reactive protein, and peripheral smear. The ocular examination was carried out in the retina clinic or at the NICU by a single examiner under aseptic precautions. Examinations followed a standardized protocol based on the International Classification of ROP (ICROP) criteria. While the use of a single observer ensured consistency in interpretation and staging, the absence of interobserver comparison represents a methodological limitation. Patients' pupils were dilated using 2.5% phenylephrine and 0.5% tropicamide. Initially, the anterior segment of the eye was examined to assess the presence of tunica vasculosa lentis, pupillary dilation, and lens/media clarity. This was followed by an examination of the posterior pole with the help of a +20.0 D lens and scleral depression to look for plus disease and then a sequential examination of all clock hours of the peripheral retina.

The first examination was conducted at 31 post-menstrual weeks or at four weeks of life, whichever came later. Afterwards, examinations were performed every two weeks or earlier if needed, until the retina was completely vascularized. Once complete vascularization was confirmed, patients were followed up at longer intervals (e.g., every 1-3 months) to monitor for any late-onset complications or recurrence, as per standard clinical protocols. All infants treated for severe ROP (n=30) were followed up for a minimum of three months post-treatment. During this period, regression of neovascularization and anatomical stabilization were documented in 85% of cases. No recurrence requiring retreatment was observed during this follow-up. However, visual function (e.g., fixation behavior, tracking) was not systematically assessed, and no retinal imaging (e.g., RetCam) was performed due to resource limitations.

The main outcome measured was the occurrence of any ROP in either eye, classified according to the ICROP [4]. The infants were divided into two groups based on the occurrence and severity of ROP. Group 1 (ROP not requiring treatment) included stage 1, 2, and 3 ROP that did not meet the threshold criteria, while group 2 (severe ROP requiring treatment) included threshold ROP and stage 4 and 5 disease. Threshold ROP was defined as the presence of at least five contiguous or eight cumulative clock hours of stage 3 ROP with plus disease in zone I or II, based on criteria from the ETROP study [5]. All infants in group 2 were treated with laser photocoagulation within 72 hours of diagnosis as per standard institutional protocol. Maternal variables such as antenatal steroid use, maternal hypertension or preeclampsia, and serial neonatal hemoglobin levels were not included in the analysis due to incomplete or inconsistent documentation across medical records, particularly for infants referred from peripheral centers.

The data were entered into an MS Excel sheet (Microsoft Corporation, Redmond, Washington, United States). Statistical software such as IBM SPSS Statistics for Windows, Version 25.0 (IBM Corp., Armonk, New York, United States), was used for all statistical analyses. Categorical (nominal) variables were presented as numbers and percentages and were analyzed using the chi-squared test or Fisher's exact test, as appropriate. Fisher's exact test was used when one of the cells in a 2x2 contingency table has an expected value of <5. Continuous variables were expressed as mean and standard deviation (SD) and were compared using independent samples t-tests between the two groups. A p-value of <0.05 was considered statistically significant.

## Results

A total of 300 preterm infants meeting the inclusion criteria were enrolled in the study over the three-year period. The demographic and clinical characteristics of the study population are summarized in Table 1.

**Table 1:** Demographic and clinical characteristics of the study population ROP: retinopathy of prematurity

Characteristics	Overall (n=300)	No ROP (n=180; 60%)	Any ROP (n= 90; 30%)	Severe ROP (n=30; 10%)
Gestational age (weeks)	32.1±2.4	32.8±2.1	31.0±2.3	29.8±1.5
Birth weight (grams)	1500±400	1600±350	1400±450	1200±250
Male (%)	56.7	55.2	60	63.3
Oxygen therapy >7 days (%)	55	40	70	85
Multiple pregnancies (%)	25	20	30	40
Blood transfusions (%)	35	30	40	55

The mean gestational age was 32.1±2.4 weeks, and the mean birth weight was 1500±400 grams. The study cohort comprised 170 males (56.7%) and 130 females (43.3%).

Incidence of ROP

Out of the 300 infants screened, 90 (30%) developed some stage of ROP. Of these, 60 infants (20%) had mild ROP (stage 1, 2, or 3 below threshold), while 30 infants (10%) developed severe ROP (threshold stage 3, 4, or 5) requiring treatment. All infants with severe ROP (n=30; 10%) were treated with laser photocoagulation. After the treatment, ROP regressed in all the infants.

Risk factors

The occurrence of ROP was significantly associated with several risk factors (Table 2).

**Table 2:** Risk factors for severe ROP OR were calculated using 2x2 contingency tables. Gestational age and birth weight were analyzed as continuous variables and not included in OR calculations. ROP: retinopathy of prematurity; OR: odds ratio; CI: confidence interval

Risk factors	Mild/no ROP	Severe ROP	P-value	OR (95% CI)
Gestational age	32.8±2.1	29.8±1.5	<0.001	-
Birth weight (grams)	1600±350	1200±250	<0.001	-
Oxygen therapy >7 days (%)	40	85	<0.001	8.9 (3.6-22.1)
Sepsis (%)	30	70	<0.001	5.5 (2.3-12.8)
Multiple pregnancies (%)	20	40	<0.020	2.7 (1.2-6.2)
Blood transfusions (%)	30	55	<0.015	2.8 (1.2-6.3)

Infants with severe ROP (n=30; 10%) requiring treatment had a lower mean gestational age (29.8±1.5 weeks) compared to those with mild ROP (n=60; 20%) or no ROP (32.8±2.1 weeks) (p<0.001). Similarly, the mean birth weight was significantly lower in the severe ROP (n=30; 10%) group (1200±250 grams) compared to the mild (n=60; 20%)/no ROP group (1600±350 grams) (p<0.001).

Other significant risk factors for ROP included prolonged oxygen therapy, sepsis, multiple pregnancies (χ²=6.3; df=1; p<0.020; φ=0.14), and blood transfusions (χ²=8.74; df=1; p<0.015; φ=0.17). Specifically, 85% of infants with severe ROP (n=30; 10%) had received oxygen therapy for more than seven days, compared to 40% in the mild (n=60; 20%)/no ROP group (χ²=25.6; df=1; p<0.001; φ=0.29). Sepsis was present in 70% of the severe ROP group versus 30% in the mild/no ROP group (χ²=19.25; df=1; p<0.001; φ=0.25).On multivariate analysis of the various risk factors in the ROP, gestational age, oxygen therapy, sepsis, and blood transfusion are independently associated with severe ROP (Table 3).

**Table 3:** Multivariate analysis of the risk factors On multivariate analysis, oxygen therapy >7 days, sepsis, birth weight, and gestational age remained statistically significant independent predictors of severe ROP. Multiple pregnancies did not retain significance after adjusting for other variables. Variables with p<0.05 are statistically significant. ROP: retinopathy of prematurity; OR: odds ratio; CI: confidence interval

Predictors	Adjusted OR	95% CI	P-value
Gestational age	0.75	0.62-0.89	0.001
Birth weight (grams)	0.998	0.996-1.000	0.047
Oxygen therapy >7 days (%)	3.5	1.6-7.8	0.002
Sepsis (%)	2.9	1.3-6.4	0.008
Multiple pregnancies (%)	1.7	0.8-3.9	0.152
Blood transfusions (%)	2.1	1.0-4.5	0.043

Clinical outcomes

All infants diagnosed with threshold ROP or worse were treated with laser photocoagulation within 72 hours of diagnosis. Post-treatment follow-up showed that 85% (n=77) of these infants had favorable anatomical outcomes, with regression of ROP and no progression to retinal detachment. However, 15% (n=13) of treated infants developed complications, including macular dragging and retinal detachment, leading to significant visual impairment.

The favorable anatomical outcomes were defined as regression of active neovascularization with no signs of retinal detachment, macular dragging, or other structural complications on subsequent examinations.

Three infants (10%) developed macular dragging, and two infants (5%) progressed to retinal detachment despite timely laser photocoagulation. All five infants had stage 3+ROP located in zone I or posterior zone II. Prolonged oxygen therapy (>7 days) and sepsis were common risk factors identified in all five cases.

In our study cohort, no cases of aggressive posterior ROP (APROP) or hybrid ROP were diagnosed during the screening period.

## Discussion

This study provides valuable insights into the epidemiology, risk factors, and clinical outcomes of ROP among preterm infants in central India [1]. This study adds to the limited epidemiological data on ROP from central India. By reporting an overall ROP incidence of 30%, with 10% requiring treatment, it helps quantify the burden of disease in this region. The detailed analysis of gestational age, birth weight, and systemic risk factors such as sepsis, oxygen therapy, and multiple gestations provides an understanding of the distribution and determinants of ROP in this neonatal population. Moreover, by comparing these findings with studies from other regions of India and Asia, the study highlights regional variability, underscoring the need for region-specific screening strategies and epidemiological surveillance. Such insights are crucial for healthcare planning and targeted neonatal care improvements in similar resource-limited settings. The findings highlight the significant burden of ROP in this region, with 30% (90 out of 300) of the screened infants developing some stage of the disease and 10% (n=9) requiring treatment for severe ROP. These results align with global trends, indicating that ROP remains a major cause of childhood blindness, particularly in regions with high rates of preterm births [1]. The incidence of ROP in our cohort is comparable to other studies conducted in developing countries. A study by Sherief et al. has shown in their study on 202 infants that the incidence of any stage ROP was 32.2% [6]. A hospital-based study conducted by Deb et al. showed that the incidence of ROP over three years was 28.7% [7]. Ahuja et al., in their prospective, observational cohort study involving babies at risk of ROP conducted in five NICUs, identified that 32.6% of babies have severe ROP [8]. Bas et al. found that the overall incidence of ROP at any stage was 27% in their study on TR-ROP [4]. The incidence of ROP reported by Xu et al. was much less than in our study (17.8% vs. 30 %) [9]. Srimanan and Kitsirilarp have also reported that the incidence of ROP in their retrospective study of 10 years was 13.6% which is less than our study [10]. Shah et al. in their study on very low birth weight infants have shown the incidence of ROP to the tune of 29.2% [11]. A retrospective analysis of records of preterm neonates from the Asian subcontinent by Yau et al. has shown that the incidence of ROP is 18.5% [12]. The study conducted by Bastola et al. in Nepal reported that more than half study subjects had ROP in at least one eye [13]. The variation in the reported percentage of ROP may be attributed to differences in cohort definition and timing of screening. Our study population included neonates who were less than 34 weeks of gestational age and those babies with a birth weight of less than 2000 grams, as well as babies who were between 34 and 36 weeks of gestational age with risk factors and were admitted to the NICU due to various risk factors.

Key findings and implications

Incidence and Risk Factors

Our data revealed that lower gestational age and birth weight are critical determinants of ROP severity. Infants with severe ROP had a mean gestational age of 29.8 weeks, the youngest being 26 weeks, and a mean birth weight of 1200 grams, significantly lower than those with mild or no ROP. These findings underscore the importance of gestational age and birth weight as primary predictors of ROP, consistent with previous research. These results were replicated by many other previously done studies. In the study done by Mamta et al., more than half neonates screened for ROP were delivered at 33-34 weeks, followed by 30-32 weeks [14]. In another Indian study by Deb et al., the mean gestational age for the study group was 30.8±2.3 weeks [7]. In a recent Ethiopian study, the mean gestational age was 32.4 weeks, ranging from 26 to 34 weeks [6]. This same result was also reflected in our study. The gestational age in the study by Bastola et al. also had results similar to ours, with a mean of 30.45±2.44 weeks [13]. In a study conducted in North India, the mean period of gestation was also similar to our study, being 31.3±2.8 weeks, though the earliest delivery was at 20 weeks [15]. The study by Mukherjee et al. also reported a comparable mean age of gestation in their study, which was 32.5±2.8 weeks [16]. The Tamil Nadu study recorded a mean gestational age of 30.68±2.84 weeks in their study with a range of 26-36 weeks [7]. However, in a study conducted by Hanif et al., the mean gestational age was lower, as compared to our study, with 28.27±1.79 weeks, whereas the majority of them were between 25 and 28 weeks of gestation [17].

Prolonged Oxygen Therapy and Sepsis

Prolonged oxygen therapy emerged as a significant risk factor, with 85% of infants with severe ROP receiving oxygen for more than seven days. This supports the well-documented association between excessive oxygen exposure and ROP development. The primary explanation lies in the fact that premature infants with lower gestational age and birth weight possess underdeveloped retinas, thus rendering them more susceptible to encountering risk factors associated with ROP, such as prolonged exposure to high concentrations of oxygen and increased vulnerability to infections [18]. Additionally, sepsis was prevalent in 70% of infants with severe ROP, highlighting the role of systemic infections in exacerbating retinal vascular proliferation. Systemic infections contribute to the pathogenesis of ROP by increasing oxidative stress, promoting pro-inflammatory cytokines, and enhancing vascular endothelial growth factor (VEGF) expression, all of which drive aberrant retinal neovascularization. This association has also been demonstrated in a meta-analysis by Xu et al., reinforcing the need for stringent infection control measures in NICUs [9]. Mehmet et al. found an association between apnea and oxygen therapy with the development of ROP [19]. This reflects a combined effect of intermittent hypoxia and increased oxygen exposure. Apneic episodes may necessitate high-flow or prolonged oxygen therapy, both of which contribute to the dysregulation of retinal vascular development. Thus, apnea may serve as a marker for greater oxygen instability, compounding the risk of ROP. Wallace et al. [20] highlighted that the use of high-flow oxygen via CPAP was associated with increased risk of severe ROP, and Karna et al. [21] confirmed that longer duration of oxygen therapy was an independent predictor of ROP development. These findings emphasize the importance of tightly regulated oxygen administration in NICU settings. Consistent with our findings, Gupta et al. indicated that oxygen, sepsis, and apnea were critical independent risk factors [22]. In the multicentric study conducted in Turkey, lower birth weight, smaller gestational age, total days on oxygen, late-onset sepsis, frequency of red blood cell transfusion, and relative weight gain were identified as independent risk factors for severe ROP but this study was done only in a cohort of infants ≤1500 g [4].

Multiple Pregnancies and Blood Transfusions

Our study also identified multiple pregnancies and blood transfusions as significant risk factors for ROP. Infants from multiple pregnancies had a higher incidence of severe ROP, possibly due to shared intrauterine growth restrictions and complications [23]. Out of 75 multiple pregnancies, 30 neonates developed any ROP (40%). Sood et al. identified multiple gestations as an independent risk factor for the development of ROP [24]. However, Rao et al. have found the incidence of ROP as 16.36% which is much less than our study [25]. Similarly, Sathar et al. have also found that multiple births were not a risk factor for the development of ROP [26]. However, Palmer et al. have shown that the likelihood of developing threshold ROP is 36% higher in multiple pregnancies [27]. Multiple gestation is not an independent pathophysiological risk factor but is commonly associated with prematurity-related complications, such as low birth weight, sepsis, and prolonged oxygen use, that increase ROP risk. Therefore, studies with different population characteristics and control of confounders may report varying results. 

Blood transfusions, often necessary in preterm infants, were more common in those with severe ROP, suggesting that transfusion-related factors may contribute to ROP pathogenesis. Bastola et al. and Nguyen et al. have also found blood transfusion as a significant risk factor for the development of ROP [13,28]. The mechanism of ROP in these cases could be due to iron overload from repeated blood transfusions, oxidative stress, and inflammation disrupting normal retinal vascular development [28]. These mechanisms warrant further investigation to optimize transfusion practices in high-risk preterm neonates.

Clinical Outcomes and Treatment Efficacy

The clinical outcomes of infants treated for severe ROP in our study were generally favorable, with 85% showing regression of the disease post-laser photocoagulation. However, 15% of treated infants developed complications such as macular dragging and retinal detachment, leading to significant visual impairment. The occurrence of macular dragging and retinal detachment in our cohort was limited to infants with posterior ROP (zone I/II) and stage 3+ disease, consistent with prior studies. These infants also had prolonged oxygen exposure and systemic infections, underscoring the aggressive nature of posterior ROP and the need for close monitoring and timely intervention [27-29]. These results emphasize the effectiveness of timely intervention but also highlight the need for ongoing follow-up and management of potential complications.

Regional Implications and Recommendations

The high prevalence of ROP in central India underscores the need for region-specific strategies to prevent and manage this condition. Our study's findings suggest several key areas for intervention.

Enhanced screening programs: Implementing comprehensive ROP screening protocols in NICUs across central India is crucial for early detection and timely treatment.

Optimized oxygen management: Developing and adhering to stringent guidelines for oxygen therapy in preterm infants can help mitigate the risk of ROP. Continuous training and monitoring of healthcare providers are essential.

Infection control: Strengthening infection control measures and ensuring prompt treatment of sepsis in NICUs can reduce the incidence of ROP associated with systemic infections.

Parental education and follow-up: Educating parents about the importance of follow-up eye examinations and the potential risks of ROP can enhance adherence to screening and treatment protocols.

While national and international guidelines for ROP screening (such as those from NNF, ETROP, and ICROP) provide a standardized framework, understanding the local epidemiology is essential for contextual adaptation and implementation. Our study highlights that in central India, risk factors such as prolonged unblended oxygen therapy, higher rates of neonatal sepsis, and late referral from peripheral centers are particularly prevalent. These insights underscore the need for earlier screening initiation in high-risk infants, strengthening of peripheral NICUs, and targeted training for timely referral.

Furthermore, identifying the high incidence (30%) and severity (10% requiring treatment) in this cohort supports the importance of maintaining a high index of suspicion even among infants near the screening thresholds (e.g., 34-36 weeks of gestation with comorbidities). Thus, local epidemiological profiling helps refine screening protocols, optimize resource allocation, and improve outcomes through earlier detection and tailored intervention strategies.

Limitations

This study has several limitations that should be acknowledged. First, as a single-center, hospital-based study conducted at a tertiary care institution in central India, the findings may not be generalizable to other settings, particularly rural areas or centers with different standards of neonatal care. The study included only preterm infants admitted to the NICU, which may introduce selection bias, as neonates born outside or managed in less specialized settings were not evaluated. Additionally, while ROP was monitored until complete retinal vascularization or post-treatment regression, the study did not include long-term follow-up of visual or neurodevelopmental outcomes, which are crucial to fully assess the impact of ROP. The district-wise comparison and the maternal variables were beyond the scope of the study. 

Another limitation is that all fundus examinations were performed by a single observer. Although this ensured consistency, it may have introduced observer bias and precluded assessment of interobserver variability or validation. While standardized techniques and classification criteria (ICROP) were followed by an experienced retinal specialist, the lack of cross-validation remains a limitation and should be addressed in future multicenter studies. Infants with incomplete records, particularly missing data on gestational age or birth weight, were excluded from the analysis, which could have influenced the incidence and risk factor estimation. Furthermore, some potentially important confounders, such as maternal health status, antenatal steroid administration, or genetic factors, were not assessed. Lastly, the number of infants with severe ROP requiring treatment was relatively small (n=30), which may limit the statistical power to detect less common but clinically significant associations. Retinal imaging using wide-field digital photography was not available during the study period.

Diagnosis was based solely on indirect ophthalmoscopy by a single examiner. While this method is clinically accepted, the lack of imaging could have limited our ability to detect early or atypical cases of APROP or hybrid ROP, which are often subtle and rapidly progressive. This represents a diagnostic limitation and highlights the need for incorporating retinal imaging, especially in resource-limited settings, to improve the detection of posterior disease.

Another limitation was not using anti-VEGF. This mode of therapy is increasingly used in cases involving zone 1 or APROP. However, during the study period at our institution, laser photocoagulation remained the primary modality of treatment, as per the prevailing institutional protocol and available infrastructure. Anti-VEGF therapy was not administered due to resource constraints and concerns about systemic safety in our setting.

The impact of utilizing oxygen blenders and the development of ROP represent the future direction of the study. 

Although anemia and low hemoglobin levels are recognized contributors to ROP pathogenesis, primarily through their role in retinal hypoxia and the need for blood transfusions, our study did not include hemoglobin levels in the analysis. This was due to inconsistent documentation and variability in the timing of hemoglobin measurements across cases, especially among infants referred from peripheral centers. As a result, a standardized and meaningful comparison was not feasible. We acknowledge this as a limitation and recommend that future prospective studies incorporate serial hemoglobin monitoring to better evaluate the association between anemia and the development or progression of ROP.

A key limitation of our study is the absence of long-term visual outcome data, such as refractive error, fixation stability, or functional vision assessments. Additionally, while all infants were followed for at least three months post-laser, longer-term follow-up to monitor for late recurrence or visual development was not performed. Future studies incorporating structured visual function testing and digital imaging may offer more comprehensive outcome data.

## Conclusions

This study highlights the significant burden of ROP among preterm infants in central India and identifies key risk factors contributing to its development. Lower gestational age, low birth weight, prolonged oxygen therapy, and blood transfusion are critical determinants of ROP severity. Early detection through enhanced screening programs and timely intervention remains paramount in preventing adverse outcomes. Our findings underscore the need for region-specific strategies and improved neonatal care practices to mitigate the risk of ROP and reduce its impact on childhood blindness in central India.

## References

[1] Gilbert C, Fielder A, Gordillo L, Quinn G, Semiglia R, Visintin P, Zin A (2005). Characteristics of infants with severe retinopathy of prematurity in countries with low, moderate, and high levels of development: implications for screening programs. Pediatrics.

[2] Parchand SM, Gangwe A, Agrawal D (2021). Commentary: retinopathy of prematurity: where do we stand?. Indian J Ophthalmol.

[3] Chandra P, Chawla D, Deorari AK (2020). Screening and management of retinopathy of prematurity. J Neonatol.

[4] Bas AY, Demirel N, Koc E, Ulubas Isik D, Hirfanoglu İM, Tunc T (2018). Incidence, risk factors and severity of retinopathy of prematurity in Turkey (TR-ROP study): a prospective, multicentre study in 69 neonatal intensive care units. Br J Ophthalmol.

[5] (2003). Revised indications for the treatment of retinopathy of prematurity: results of the early treatment for retinopathy of prematurity randomized trial. Arch Ophthalmol.

[6] Sherief ST, Taye K, Teshome T, Demtse A, Gilbert C (2023). Retinopathy of prematurity among infants admitted to two neonatal intensive care units in Ethiopia. BMJ Open Ophthalmol.

[7] Deb D, Annamalai R, Muthukumar M (2023). Incidence, risk factors, progression, and involution in retinopathy of prematurity at a tertiary care center in South India. Oman J Ophthalmol.

[8] Ahuja AA, V Reddy YC, Adenuga OO, Kewlani D, Ravindran M, Ramakrishnan R (2018). Risk factors for retinopathy of prematurity in a district in South India: a prospective cohort study. Oman J Ophthalmol.

[9] Xu Y, Zhou X, Zhang Q (2013). Screening for retinopathy of prematurity in China: a neonatal units-based prospective study. Invest Ophthalmol Vis Sci.

[10] Srimanan W, Kitsirilarp W (2023). The incidence and risk factors associated with the retinopathy of prematurity in a tertiary hospital using 10-year retrospective data. Ann Clin Epidemiol.

[11] Shah VA, Yeo CL, Ling YL, Ho LY (2005). Incidence, risk factors of retinopathy of prematurity among very low birth weight infants in Singapore. Ann Acad Med Singap.

[12] Yau GS, Lee JW, Tam VT (2016). Incidence and risk factors of retinopathy of prematurity from 2 neonatal intensive care units in a Hong Kong Chinese population. Asia Pac J Ophthalmol (Phila).

[13] Bastola P, Parchand SM, Gangwe AB, Bastola S, Agrawal D (2023). Retinopathy of prematurity among preterm newborn admitted to the neonatal care unit in a tertiary care centre: a descriptive cross-sectional study. JNMA J Nepal Med Assoc.

[14] Mamta C, Janardan S, Nisha D, Meghna S, Harish D (2023). Incidence and prevalence of retinopathy of prematurity in a tertiary care centre of North India. Maedica (Bucur).

[15] Tekchandani U, Katoch D, Dogra MR (2021). Five-year demographic profile of retinopathy of prematurity at a tertiary care institute in North India. Indian J Ophthalmol.

[16] Mukherjee S, Saha AK, Das P, Sen D, Bhar S (2020). Retinopathy of prematurity in a level II neonatal care unit of a district of West Bengal: a retrospective analysis of 5 years. Indian J Public Health.

[17] Hanif M, Ariff S, Ansar A, Ahmed K, Hussain AS (2020). Characteristics of preterm with sight threatening retinopathy of prematurity. J Ayub Med Coll Abbottabad.

[18] Chen J, Smith LE (2007). Retinopathy of prematurity. Angiogenesis.

[19] Mehmet S, Fusun A, Sebnem C (2011). One-year experience in the retinopathy of prematurity: frequency and risk factors, short-term results and follow-up. Int J Ophthalmol.

[20] Wallace DK, Veness-Meehan KA, Miller WC (2007). Incidence of severe retinopathy of prematurity before and after a modest reduction in target oxygen saturation levels. J AAPOS.

[21] Karna P, Muttineni J, Angell L, Karmaus W (2005). Retinopathy of prematurity and risk factors: a prospective cohort study. BMC Pediatr.

[22] Gupta VP, Dhaliwal U, Sharma R, Gupta P, Rohatgi J (2004). Retinopathy of prematurity - risk factors. Indian J Pediatr.

[23] Bahmani T, Karimi A, Rezaei N, Daliri S (2022). Retinopathy prematurity: a systematic review and meta-analysis study based on neonatal and maternal risk factors. J Matern Fetal Neonatal Med.

[24] Sood V, Chellani H, Arya S, Guliani BP (2012). Changing spectrum of retinopathy of prematurity (ROP) and variations among siblings of multiple gestation. Indian J Pediatr.

[25] Rao KA, Purkayastha J, Hazarika M, Chaitra R, Adith KM (2013). Analysis of prenatal and postnatal risk factors of retinopathy of prematurity in a tertiary care hospital in South India. Indian J Ophthalmol.

[26] Sathar A, Shanavas A, Girijadevi PS, Jasmin LB, Kumar S, Pillai RK (2018). Risk factors of retinopathy of prematurity in a tertiary care hospital in South India. Clin Epidemiol Glob Health.

[27] Palmer EA, Flynn JT, Hardy RJ, Phelps DL, Phillips CL, Schaffer DB, Tung B (2020). Incidence and early course of retinopathy of prematurity. Ophthalmology.

[28] Nguyen TT, Bui VT, Pham VP, Pham TN (2022). Retinopathy of prematurity: a study of incidence and risk factors in a tertiary hospital in Vietnam. Clin Ophthalmol.

[29] Strelnikov JI, Rao R, Majidi S, Lueder G, Lee A, Reynolds MM (2024). Retinopathy of prematurity screening: prevalence and risk factors of ophthalmic complications in non-treated preterm infants. Eye (Lond).

